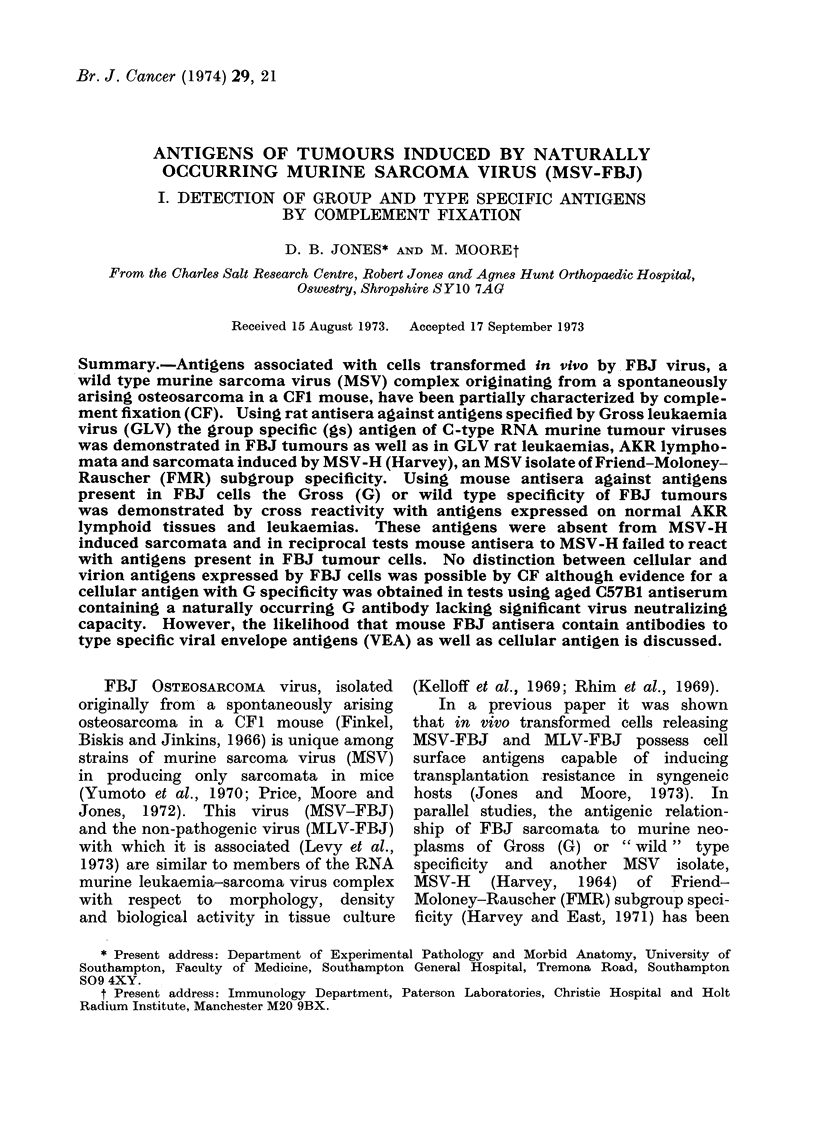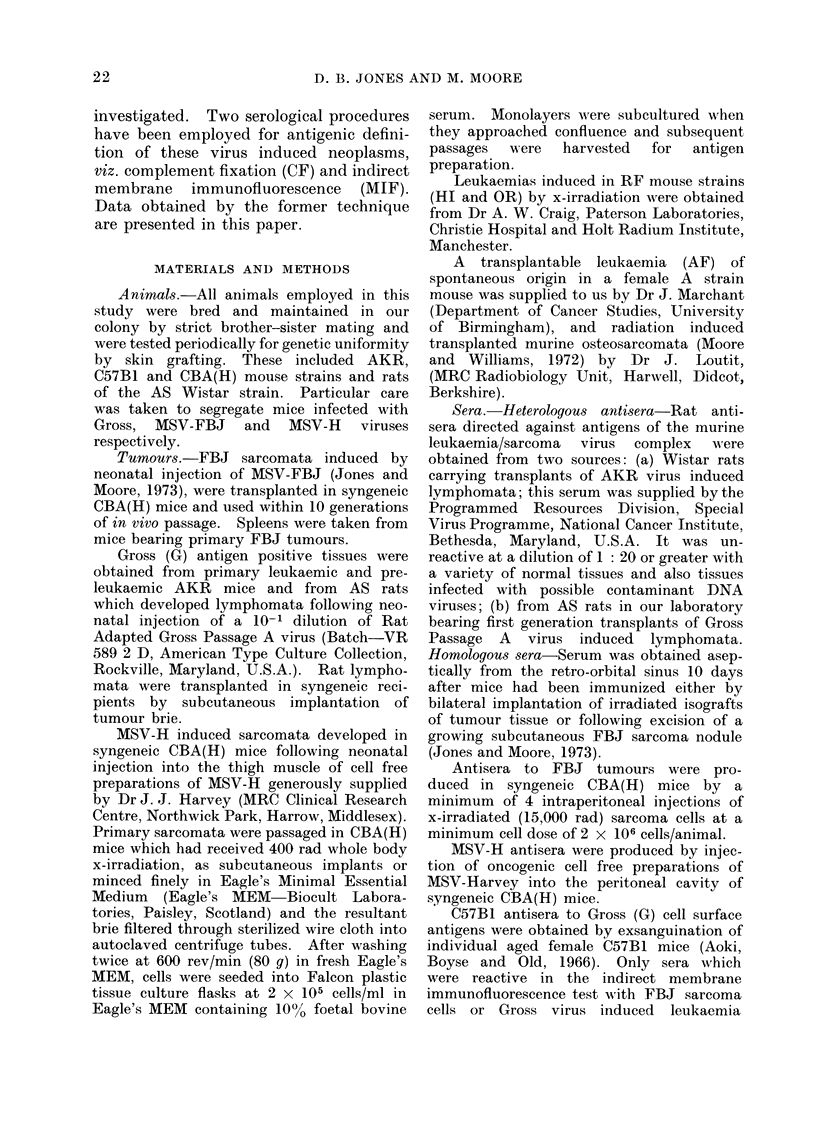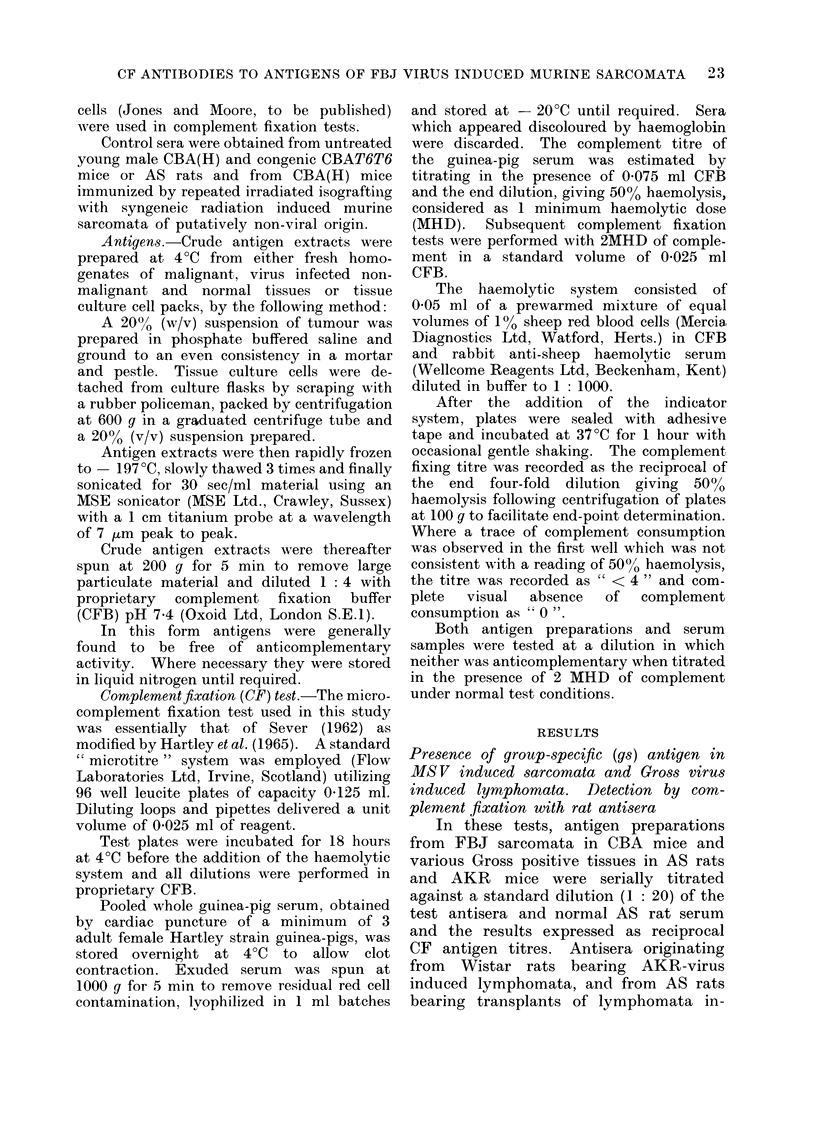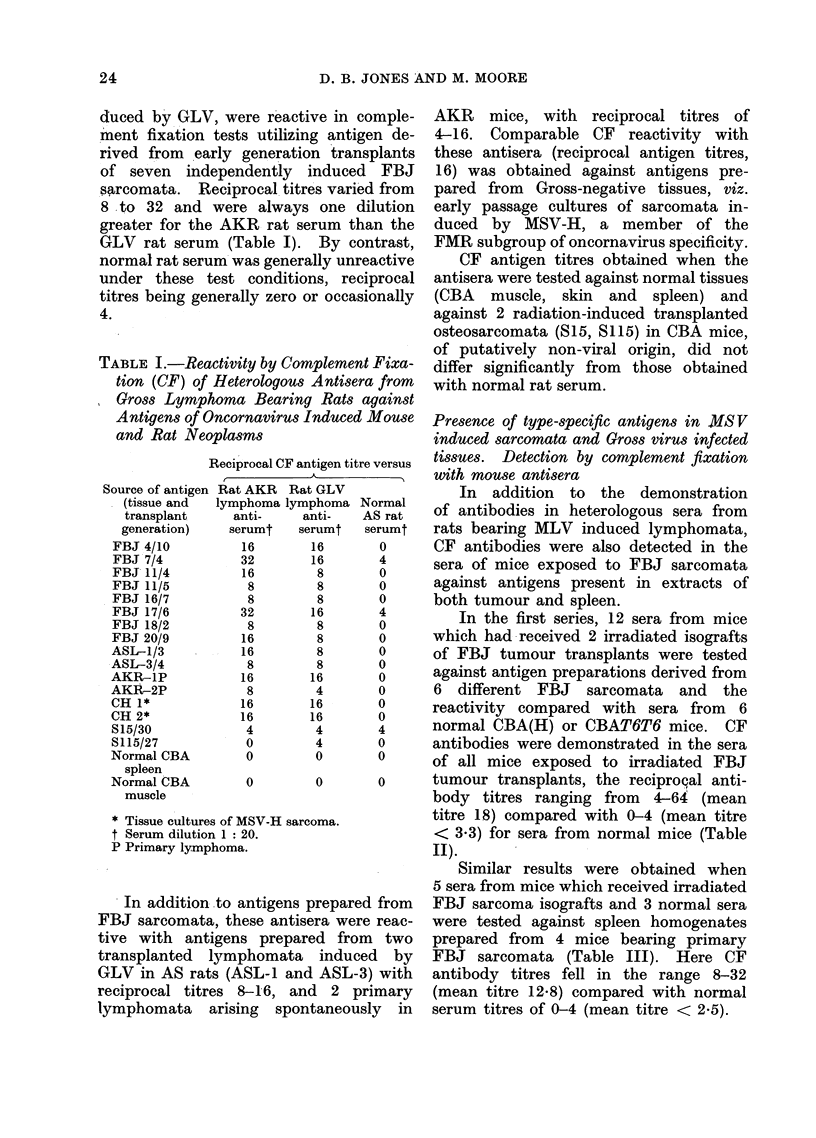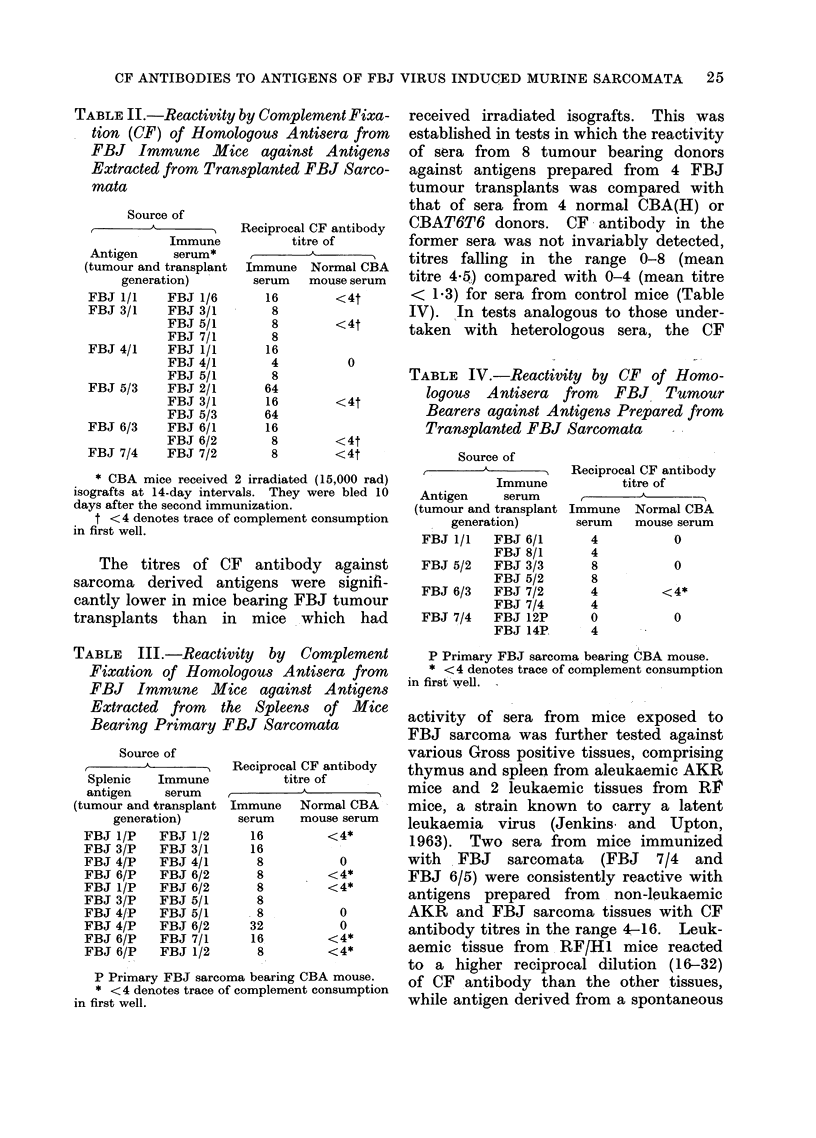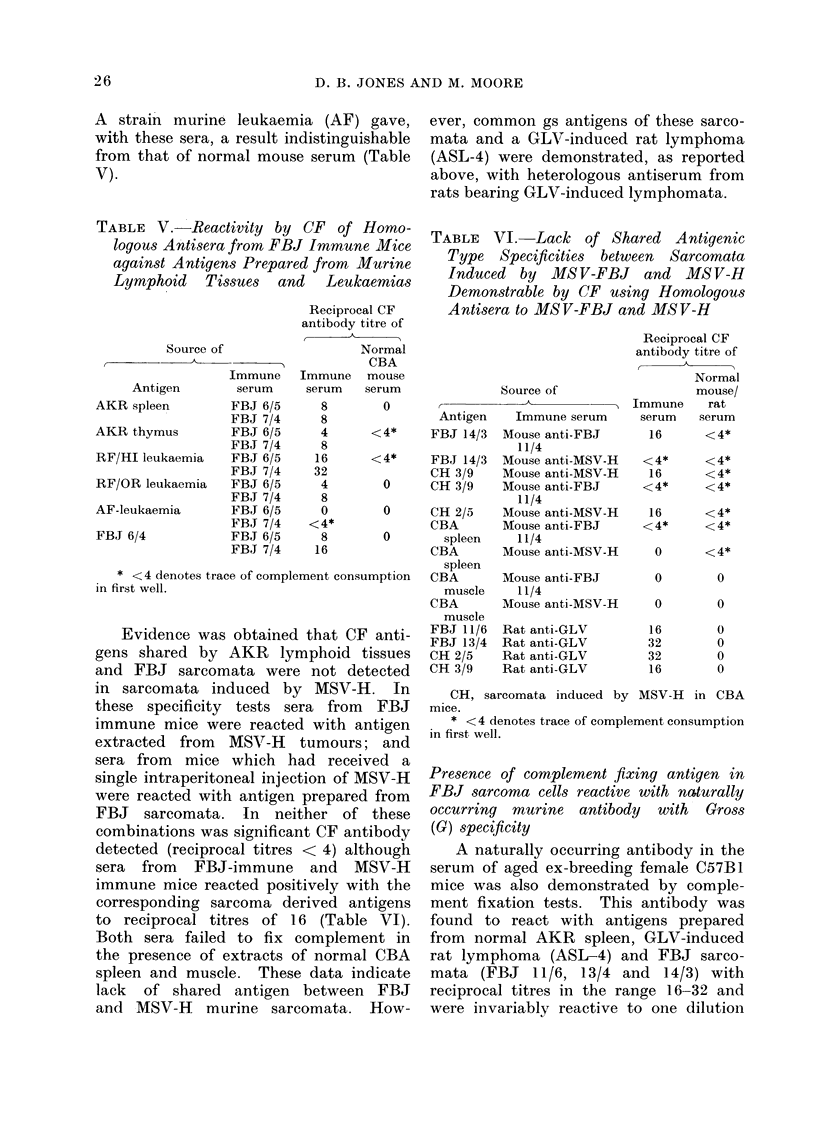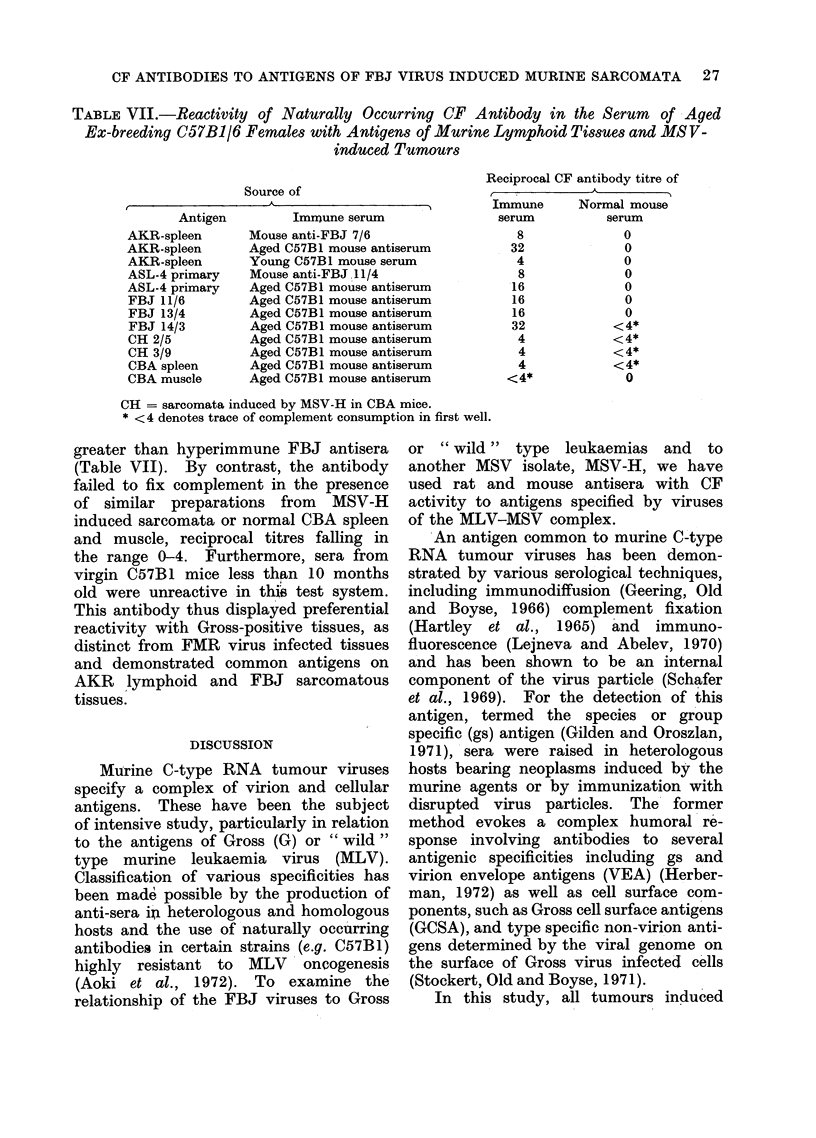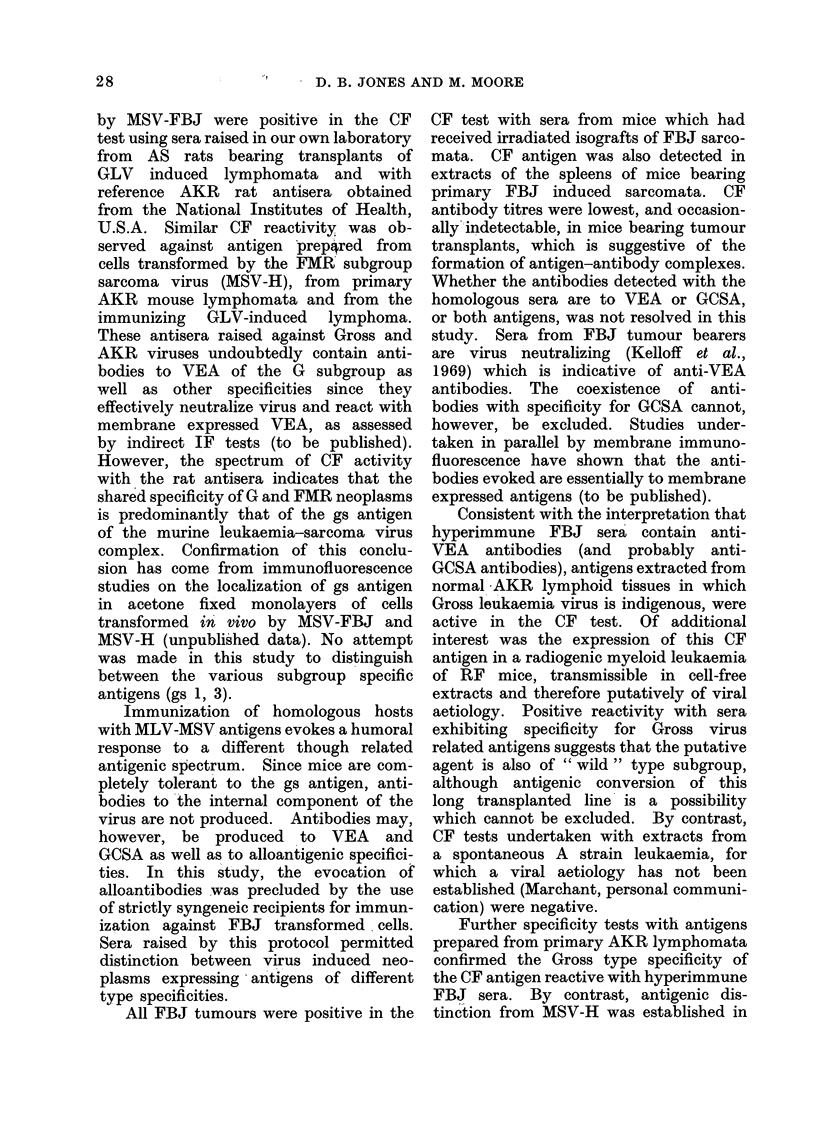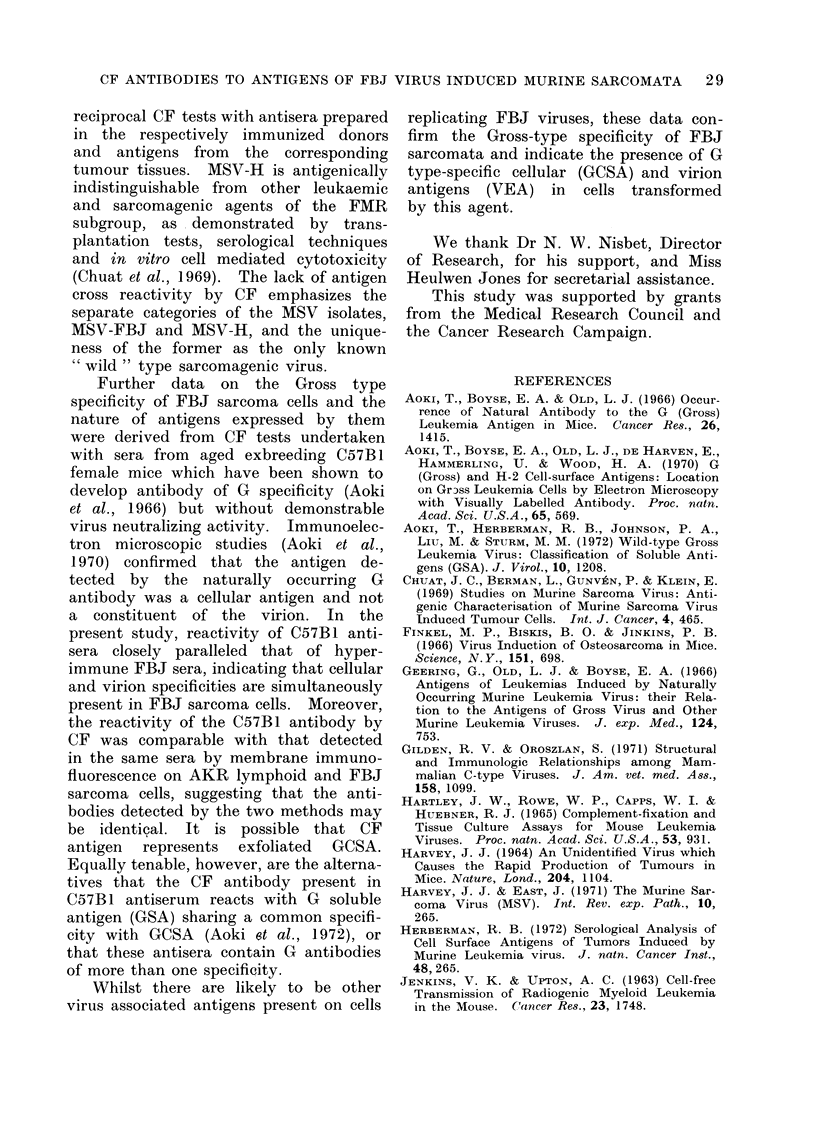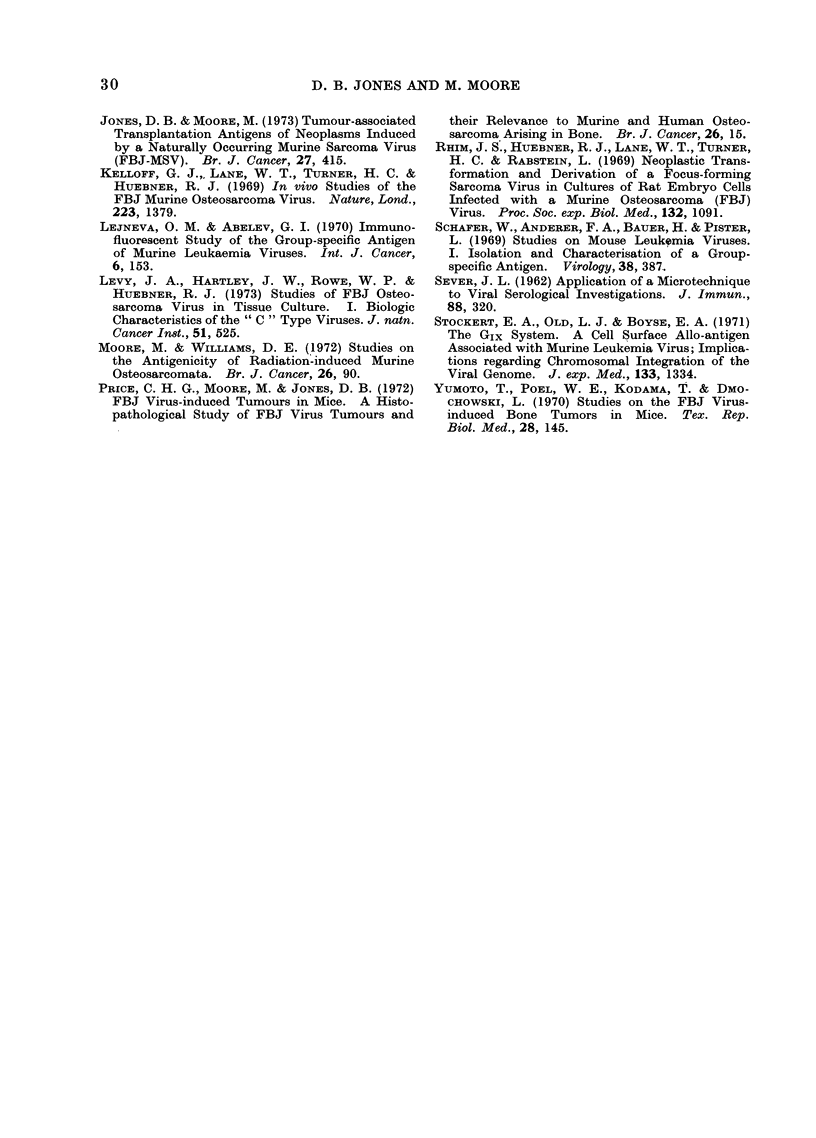# Antigens of Tumours Induced by Naturally Occurring Murine Sarcoma Virus (MSV-FBJ)

**DOI:** 10.1038/bjc.1974.3

**Published:** 1974-01

**Authors:** D. B. Jones, M. Moore

## Abstract

Antigens associated with cells transformed *in vivo* by FBJ virus, a wild type murine sarcoma virus (MSV) complex originating from a spontaneously arising osteosarcoma in a CF1 mouse, have been partially characterized by complement fixation (CF). Using rat antisera against antigens specified by Gross leukaemia virus (GLV) the group specific (gs) antigen of C-type RNA murine tumour viruses was demonstrated in FBJ tumours as well as in GLV rat leukaemias, AKR lymphomata and sarcomata induced by MSV-H (Harvey), an MSV isolate of Friend-Moloney-Rauscher (FMR) subgroup specificity. Using mouse antisera against antigens present in FBJ cells the Gross (G) or wild type specificity of FBJ tumours was demonstrated by cross reactivity with antigens expressed on normal AKR lymphoid tissues and leukaemias. These antigens were absent from MSV-H induced sarcomata and in reciprocal tests mouse antisera to MSV-H failed to react with antigens present in FBJ tumour cells. No distinction between cellular and virion antigens expressed by FBJ cells was possible by CF although evidence for a cellular antigen with G specificity was obtained in tests using aged C57B1 antiserum containing a naturally occurring G antibody lacking significant virus neutralizing capacity. However, the likelihood that mouse FBJ antisera contain antibodies to type specific viral envelope antigens (VEA) as well as cellular antigen is discussed.


*Br. J. Cancer* (1974) **29**, 21

### ANTIGENS OF TUMOURS INDUCED BY NATURALLY OCCURRING MURINE SARCOMA VIRUS (MSV-FBJ)

### I. DETECTION OF GROUP AND TYPE SPECIFIC ANTIGENS BY COMPLEMENT FIXATION

D. B. JONES* AND M. MOORE†

*From the Charles Salt Research Centre, Robert Jones and Agnes Hunt Orthopaedic Hospital, Oswestry, Shropshire SY10 7AG*

Received 15 August 1973. Accepted 17 September 1973

**Summary.—Antigens associated with cells transformed *in vivo* by FBJ virus, a wild type murine sarcoma virus (MSV) complex originating from a spontaneously arising osteosarcoma in a CF1 mouse, have been partially characterized by complement fixation (CF). Using rat antisera against antigens specified by Gross leukaemia virus (GLV) the group specific (gs) antigen of C-type RNA murine tumour viruses was demonstrated in FBJ tumours as well as in GLV rat leukaemias, AKR lymphomata and sarcomata induced by MSV-H (Harvey), an MSV isolate of Friend–Moloney–Rauscher (FMR) subgroup specificity. Using mouse antisera against antigens present in FBJ cells the Gross (G) or wild type specificity of FBJ tumours was demonstrated by cross reactivity with antigens expressed on normal AKR lymphoid tissues and leukaemias. These antigens were absent from MSV-H induced sarcomata and in reciprocal tests mouse antisera to MSV-H failed to react with antigens present in FBJ tumour cells. No distinction between cellular and virion antigens expressed by FBJ cells was possible by CF although evidence for a cellular antigen with G specificity was obtained in tests using aged C57B1 antiserum containing a naturally occurring G antibody lacking significant virus neutralizing capacity. However, the likelihood that mouse FBJ antisera contain antibodies to type specific viral envelope antigens (VEA) as well as cellular antigen is discussed.**

FBJ OSTEOSARCOMA virus, isolated originally from a spontaneously arising osteosarcoma in a CF1 mouse (Finkel, Biskis and Jinkins, 1966) is unique among strains of murine sarcoma virus (MSV) in producing only sarcomata in mice (Yumoto *et al.*, 1970; Price, Moore and Jones, 1972). This virus (MSV–FBJ) and the non-pathogenic virus (MLV-FBJ) with which it is associated (Levy *et al.*, 1973) are similar to members of the RNA murine leukaemia–sarcoma virus complex with respect to morphology, density and biological activity in tissue culture (Kelloff *et al.*, 1969; Rhim *et al.*, 1969).

In a previous paper it was shown that *in vivo* transformed cells releasing MSV-FBJ and MLV-FBJ possess cell surface antigens capable of inducing transplantation resistance in syngeneic hosts (Jones and Moore, 1973). In parallel studies, the antigenic relationship of FBJ sarcomata to murine neoplasms of Gross (G) or " wild " type specificity and another MSV isolate, MSV-H (Harvey, 1964) of Friend–Moloney–Rauscher (FMR) subgroup specificity (Harvey and East, 1971) has been

* Present address: Department of Experimental Pathology and Morbid Anatomy, University of Southampton, Faculty of Medicine, Southampton General Hospital, Tremona Road, Southampton SO9 4XY.

† Present address: Immunology Department, Paterson Laboratories, Christie Hospital and Holt Radium Institute, Manchester M20 9BX.

investigated. Two serological procedures have been employed for antigenic definition of these virus induced neoplasms, *viz.* complement fixation (CF) and indirect membrane immunofluorescence (MIF). Data obtained by the former technique are presented in this paper.

### MATERIALS AND METHODS

*Animals.*—All animals employed in this study were bred and maintained in our colony by strict brother–sister mating and were tested periodically for genetic uniformity by skin grafting. These included AKR, C57Bl and CBA(H) mouse strains and rats of the AS Wistar strain. Particular care was taken to segregate mice infected with Gross, MSV-FBJ and MSV-H viruses respectively.

*Tumours.*—FBJ sarcomata induced by neonatal injection of MSV-FBJ (Jones and Moore, 1973), were transplanted in syngeneic CBA(H) mice and used within 10 generations of *in vivo* passage. Spleens were taken from mice bearing primary FBJ tumours.

Gross (G) antigen positive tissues were obtained from primary leukaemic and preleukaemic AKR mice and from AS rats which developed lymphomata following neonatal injection of a $10^{-1}$ dilution of Rat Adapted Gross Passage A virus (Batch—VR 589 2 D, American Type Culture Collection, Rockville, Maryland, U.S.A.). Rat lymphomata were transplanted in syngeneic recipients by subcutaneous implantation of tumour brie.

MSV-H induced sarcomata developed in syngeneic CBA(H) mice following neonatal injection into the thigh muscle of cell free preparations of MSV-H generously supplied by Dr J. J. Harvey (MRC Clinical Research Centre, Northwick Park, Harrow, Middlesex). Primary sarcomata were passaged in CBA(H) mice which had received 400 rad whole body x-irradiation, as subcutaneous implants or minced finely in Eagle's Minimal Essential Medium (Eagle's MEM—Biocult Laboratories, Paisley, Scotland) and the resultant brie filtered through sterilized wire cloth into autoclaved centrifuge tubes. After washing twice at 600 rev/min (80 *g*) in fresh Eagle's MEM, cells were seeded into Falcon plastic tissue culture flasks at $2 \times 10^5$ cells/ml in Eagle's MEM containing 10% foetal bovine serum. Monolayers were subcultured when they approached confluence and subsequent passages were harvested for antigen preparation.

Leukaemias induced in RF mouse strains (HI and OR) by x-irradiation were obtained from Dr A. W. Craig, Paterson Laboratories, Christie Hospital and Holt Radium Institute, Manchester.

A transplantable leukaemia (AF) of spontaneous origin in a female A strain mouse was supplied to us by Dr J. Marchant (Department of Cancer Studies, University of Birmingham), and radiation induced transplanted murine osteosarcomata (Moore and Williams, 1972) by Dr J. Loutit, (MRC Radiobiology Unit, Harwell, Didcot, Berkshire).

*Sera.*—*Heterologous antisera*—Rat antisera directed against antigens of the murine leukaemia/sarcoma virus complex were obtained from two sources: (a) Wistar rats carrying transplants of AKR virus induced lymphomata; this serum was supplied by the Programmed Resources Division, Special Virus Programme, National Cancer Institute, Bethesda, Maryland, U.S.A. It was unreactive at a dilution of 1 : 20 or greater with a variety of normal tissues and also tissues infected with possible contaminant DNA viruses; (b) from AS rats in our laboratory bearing first generation transplants of Gross Passage A virus induced lymphomata. *Homologous sera*—Serum was obtained aseptically from the retro-orbital sinus 10 days after mice had been immunized either by bilateral implantation of irradiated isografts of tumour tissue or following excision of a growing subcutaneous FBJ sarcoma nodule (Jones and Moore, 1973).

Antisera to FBJ tumours were produced in syngeneic CBA(H) mice by a minimum of 4 intraperitoneal injections of x-irradiated (15,000 rad) sarcoma cells at a minimum cell dose of $2 \times 10^6$ cells/animal.

MSV-H antisera were produced by injection of oncogenic cell free preparations of MSV-Harvey into the peritoneal cavity of syngeneic CBA(H) mice.

C57Bl antisera to Gross (G) cell surface antigens were obtained by exsanguination of individual aged female C57Bl mice (Aoki, Boyse and Old, 1966). Only sera which were reactive in the indirect membrane immunofluorescence test with FBJ sarcoma cells or Gross virus induced leukaemia cells (Jones and Moore, to be published) were used in complement fixation tests.

Control sera were obtained from untreated young male CBA(H) and congenic CBA*T6T6* mice or AS rats and from CBA(H) mice immunized by repeated irradiated isografting with syngeneic radiation induced murine sarcomata of putatively non-viral origin.

*Antigens*.—Crude antigen extracts were prepared at 4°C from either fresh homogenates of malignant, virus infected non-malignant and normal tissues or tissue culture cell packs, by the following method:

A 20% (w/v) suspension of tumour was prepared in phosphate buffered saline and ground to an even consistency in a mortar and pestle. Tissue culture cells were detached from culture flasks by scraping with a rubber policeman, packed by centrifugation at 600 *g* in a graduated centrifuge tube and a 20% (v/v) suspension prepared.

Antigen extracts were then rapidly frozen to — 197°C, slowly thawed 3 times and finally sonicated for 30 sec/ml material using an MSE sonicator (MSE Ltd., Crawley, Sussex) with a 1 cm titanium probe at a wavelength of 7 $\mu$m peak to peak.

Crude antigen extracts were thereafter spun at 200 *g* for 5 min to remove large particulate material and diluted 1 : 4 with proprietary complement fixation buffer (CFB) pH 7·4 (Oxoid Ltd, London S.E.1).

In this form antigens were generally found to be free of anticomplementary activity. Where necessary they were stored in liquid nitrogen until required.

*Complement fixation (CF) test*.—The micro-complement fixation test used in this study was essentially that of Sever (1962) as modified by Hartley *et al.* (1965). A standard " microtitre " system was employed (Flow Laboratories Ltd, Irvine, Scotland) utilizing 96 well leucite plates of capacity 0·125 ml. Diluting loops and pipettes delivered a unit volume of 0·025 ml of reagent.

Test plates were incubated for 18 hours at 4°C before the addition of the haemolytic system and all dilutions were performed in proprietary CFB.

Pooled whole guinea-pig serum, obtained by cardiac puncture of a minimum of 3 adult female Hartley strain guinea-pigs, was stored overnight at 4°C to allow clot contraction. Exuded serum was spun at 1000 *g* for 5 min to remove residual red cell contamination, lyophilized in 1 ml batches and stored at — 20°C until required. Sera which appeared discoloured by haemoglobin were discarded. The complement titre of the guinea-pig serum was estimated by titrating in the presence of 0·075 ml CFB and the end dilution, giving 50% haemolysis, considered as 1 minimum haemolytic dose (MHD). Subsequent complement fixation tests were performed with 2MHD of complement in a standard volume of 0·025 ml CFB.

The haemolytic system consisted of 0·05 ml of a prewarmed mixture of equal volumes of 1% sheep red blood cells (Mercia Diagnostics Ltd, Watford, Herts.) in CFB and rabbit anti-sheep haemolytic serum (Wellcome Reagents Ltd, Beckenham, Kent) diluted in buffer to 1 : 1000.

After the addition of the indicator system, plates were sealed with adhesive tape and incubated at 37°C for 1 hour with occasional gentle shaking. The complement fixing titre was recorded as the reciprocal of the end four-fold dilution giving 50% haemolysis following centrifugation of plates at 100 *g* to facilitate end-point determination. Where a trace of complement consumption was observed in the first well which was not consistent with a reading of 50% haemolysis, the titre was recorded as " $< 4$ " and complete visual absence of complement consumption as " 0 ".

Both antigen preparations and serum samples were tested at a dilution in which neither was anticomplementary when titrated in the presence of 2 MHD of complement under normal test conditions.

### RESULTS

*Presence of group-specific (gs) antigen in MSV induced sarcomata and Gross virus induced lymphomata. Detection by complement fixation with rat antisera*

In these tests, antigen preparations from FBJ sarcomata in CBA mice and various Gross positive tissues in AS rats and AKR mice were serially titrated against a standard dilution (1 : 20) of the test antisera and normal AS rat serum and the results expressed as reciprocal CF antigen titres. Antisera originating from Wistar rats bearing AKR-virus induced lymphomata, and from AS rats bearing transplants of lymphomata induced by GLV, were reactive in complement fixation tests utilizing antigen derived from early generation transplants of seven independently induced FBJ sarcomata. Reciprocal titres varied from 8 to 32 and were always one dilution greater for the AKR rat serum than the GLV rat serum (Table I). By contrast, normal rat serum was generally unreactive under these test conditions, reciprocal titres being generally zero or occasionally 4.

TABLE I.—*Reactivity by Complement Fixation (CF) of Heterologous Antisera from Gross Lymphoma Bearing Rats against Antigens of Oncornavirus Induced Mouse and Rat Neoplasms*

| Source of antigen (tissue and transplant generation) | Reciprocal CF antigen titre versus Rat AKR lymphoma anti-serum† | Rat GLV lymphoma anti-serum† | Normal AS rat serum† |
|---|---|---|---|
| FBJ 4/10 | 16 | 16 | 0 |
| FBJ 7/4 | 32 | 16 | 4 |
| FBJ 11/4 | 16 | 8 | 0 |
| FBJ 11/5 | 8 | 8 | 0 |
| FBJ 16/7 | 8 | 8 | 0 |
| FBJ 17/6 | 32 | 16 | 4 |
| FBJ 18/2 | 8 | 8 | 0 |
| FBJ 20/9 | 16 | 8 | 0 |
| ASL–1/3 | 16 | 8 | 0 |
| ASL–3/4 | 8 | 8 | 0 |
| AKR–1P | 16 | 16 | 0 |
| AKR–2P | 8 | 4 | 0 |
| CH 1* | 16 | 16 | 0 |
| CH 2* | 16 | 16 | 0 |
| S15/30 | 4 | 4 | 4 |
| S115/27 | 0 | 4 | 0 |
| Normal CBA spleen | 0 | 0 | 0 |
| Normal CBA muscle | 0 | 0 | 0 |

* Tissue cultures of MSV-H sarcoma.
† Serum dilution 1 : 20.
P Primary lymphoma.

In addition to antigens prepared from FBJ sarcomata, these antisera were reactive with antigens prepared from two transplanted lymphomata induced by GLV in AS rats (ASL-1 and ASL-3) with reciprocal titres 8–16, and 2 primary lymphomata arising spontaneously in AKR mice, with reciprocal titres of 4–16. Comparable CF reactivity with these antisera (reciprocal antigen titres, 16) was obtained against antigens prepared from Gross-negative tissues, *viz.* early passage cultures of sarcomata induced by MSV-H, a member of the FMR subgroup of oncornavirus specificity.

CF antigen titres obtained when the antisera were tested against normal tissues (CBA muscle, skin and spleen) and against 2 radiation-induced transplanted osteosarcomata (S15, S115) in CBA mice, of putatively non-viral origin, did not differ significantly from those obtained with normal rat serum.

*Presence of type-specific antigens in MSV induced sarcomata and Gross virus infected tissues. Detection by complement fixation with mouse antisera*

In addition to the demonstration of antibodies in heterologous sera from rats bearing MLV induced lymphomata, CF antibodies were also detected in the sera of mice exposed to FBJ sarcomata against antigens present in extracts of both tumour and spleen.

In the first series, 12 sera from mice which had received 2 irradiated isografts of FBJ tumour transplants were tested against antigen preparations derived from 6 different FBJ sarcomata and the reactivity compared with sera from 6 normal CBA(H) or CBA*T6T6* mice. CF antibodies were demonstrated in the sera of all mice exposed to irradiated FBJ tumour transplants, the reciprocal antibody titres ranging from 4–64 (mean titre 18) compared with 0–4 (mean titre $< 3{\cdot}3$) for sera from normal mice (Table II).

Similar results were obtained when 5 sera from mice which received irradiated FBJ sarcoma isografts and 3 normal sera were tested against spleen homogenates prepared from 4 mice bearing primary FBJ sarcomata (Table III). Here CF antibody titres fell in the range 8–32 (mean titre 12·8) compared with normal serum titres of 0–4 (mean titre $< 2{\cdot}5$).

TABLE II.—*Reactivity by Complement Fixation (CF) of Homologous Antisera from FBJ Immune Mice against Antigens Extracted from Transplanted FBJ Sarcomata*

| Source of: Antigen (tumour and transplant generation) | Source of: Immune serum* | Reciprocal CF antibody titre of: Immune serum | Reciprocal CF antibody titre of: Normal CBA mouse serum |
|---|---|---|---|
| FBJ 1/1 | FBJ 1/6 | 16 | <4† |
| FBJ 3/1 | FBJ 3/1 | 8 | |
| | FBJ 5/1 | 8 | <4† |
| | FBJ 7/1 | 8 | |
| FBJ 4/1 | FBJ 1/1 | 16 | |
| | FBJ 4/1 | 4 | 0 |
| | FBJ 5/1 | 8 | |
| FBJ 5/3 | FBJ 2/1 | 64 | |
| | FBJ 3/1 | 16 | <4† |
| | FBJ 5/3 | 64 | |
| FBJ 6/3 | FBJ 6/1 | 16 | |
| | FBJ 6/2 | 8 | <4† |
| FBJ 7/4 | FBJ 7/2 | 8 | <4† |

* CBA mice received 2 irradiated (15,000 rad) isografts at 14-day intervals. They were bled 10 days after the second immunization.

† <4 denotes trace of complement consumption in first well.

The titres of CF antibody against sarcoma derived antigens were significantly lower in mice bearing FBJ tumour transplants than in mice which had received irradiated isografts. This was established in tests in which the reactivity of sera from 8 tumour bearing donors against antigens prepared from 4 FBJ tumour transplants was compared with that of sera from 4 normal CBA(H) or CBA*T6T6* donors. CF antibody in the former sera was not invariably detected, titres falling in the range 0–8 (mean titre 4·5) compared with 0–4 (mean titre < 1·3) for sera from control mice (Table IV). In tests analogous to those undertaken with heterologous sera, the CF activity of sera from mice exposed to FBJ sarcoma was further tested against various Gross positive tissues, comprising thymus and spleen from aleukaemic AKR mice and 2 leukaemic tissues from RF mice, a strain known to carry a latent leukaemia virus (Jenkins and Upton, 1963). Two sera from mice immunized with FBJ sarcomata (FBJ 7/4 and FBJ 6/5) were consistently reactive with antigens prepared from non-leukaemic AKR and FBJ sarcoma tissues with CF antibody titres in the range 4–16. Leukaemic tissue from RF/H1 mice reacted to a higher reciprocal dilution (16–32) of CF antibody than the other tissues, while antigen derived from a spontaneous

TABLE III.—*Reactivity by Complement Fixation of Homologous Antisera from FBJ Immune Mice against Antigens Extracted from the Spleens of Mice Bearing Primary FBJ Sarcomata*

| Source of: Splenic antigen (tumour and transplant generation) | Source of: Immune serum | Reciprocal CF antibody titre of: Immune serum | Reciprocal CF antibody titre of: Normal CBA mouse serum |
|---|---|---|---|
| FBJ 1/P | FBJ 1/2 | 16 | <4* |
| FBJ 3/P | FBJ 3/1 | 16 | |
| FBJ 4/P | FBJ 4/1 | 8 | 0 |
| FBJ 6/P | FBJ 6/2 | 8 | <4* |
| FBJ 1/P | FBJ 6/2 | 8 | <4* |
| FBJ 3/P | FBJ 5/1 | 8 | |
| FBJ 4/P | FBJ 5/1 | 8 | 0 |
| FBJ 4/P | FBJ 6/2 | 32 | 0 |
| FBJ 6/P | FBJ 7/1 | 16 | <4* |
| FBJ 6/P | FBJ 1/2 | 8 | <4* |

P Primary FBJ sarcoma bearing CBA mouse.

* <4 denotes trace of complement consumption in first well.

TABLE IV.—*Reactivity by CF of Homologous Antisera from FBJ Tumour Bearers against Antigens Prepared from Transplanted FBJ Sarcomata*

| Source of: Antigen (tumour and transplant generation) | Source of: Immune serum | Reciprocal CF antibody titre of: Immune serum | Reciprocal CF antibody titre of: Normal CBA mouse serum |
|---|---|---|---|
| FBJ 1/1 | FBJ 6/1 | 4 | 0 |
| | FBJ 8/1 | 4 | |
| FBJ 5/2 | FBJ 3/3 | 8 | 0 |
| | FBJ 5/2 | 8 | |
| FBJ 6/3 | FBJ 7/2 | 4 | <4* |
| | FBJ 7/4 | 4 | |
| FBJ 7/4 | FBJ 12P | 0 | 0 |
| | FBJ 14P | 4 | |

P Primary FBJ sarcoma bearing CBA mouse.

* <4 denotes trace of complement consumption in first well.

A strain murine leukaemia (AF) gave, with these sera, a result indistinguishable from that of normal mouse serum (Table V).

TABLE V.—*Reactivity by CF of Homologous Antisera from FBJ Immune Mice against Antigens Prepared from Murine Lymphoid Tissues and Leukaemias*

| Source of | | Reciprocal CF antibody titre of | |
|---|---|---|---|
| Antigen | Immune serum | Immune serum | Normal CBA mouse serum |
| AKR spleen | FBJ 6/5 | 8 | 0 |
| | FBJ 7/4 | 8 | |
| AKR thymus | FBJ 6/5 | 4 | <4* |
| | FBJ 7/4 | 8 | |
| RF/HI leukaemia | FBJ 6/5 | 16 | <4* |
| | FBJ 7/4 | 32 | |
| RF/OR leukaemia | FBJ 6/5 | 4 | 0 |
| | FBJ 7/4 | 8 | |
| AF-leukaemia | FBJ 6/5 | 0 | 0 |
| | FBJ 7/4 | <4* | |
| FBJ 6/4 | FBJ 6/5 | 8 | 0 |
| | FBJ 7/4 | 16 | |

* <4 denotes trace of complement consumption in first well.

Evidence was obtained that CF antigens shared by AKR lymphoid tissues and FBJ sarcomata were not detected in sarcomata induced by MSV-H. In these specificity tests sera from FBJ immune mice were reacted with antigen extracted from MSV-H tumours; and sera from mice which had received a single intraperitoneal injection of MSV-H were reacted with antigen prepared from FBJ sarcomata. In neither of these combinations was significant CF antibody detected (reciprocal titres < 4) although sera from FBJ-immune and MSV-H immune mice reacted positively with the corresponding sarcoma derived antigens to reciprocal titres of 16 (Table VI). Both sera failed to fix complement in the presence of extracts of normal CBA spleen and muscle. These data indicate lack of shared antigen between FBJ and MSV-H murine sarcomata. However, common gs antigens of these sarcomata and a GLV-induced rat lymphoma (ASL-4) were demonstrated, as reported above, with heterologous antiserum from rats bearing GLV-induced lymphomata.

TABLE VI.—*Lack of Shared Antigenic Type Specificities between Sarcomata Induced by MSV-FBJ and MSV-H Demonstrable by CF using Homologous Antisera to MSV-FBJ and MSV-H*

| Source of | | Reciprocal CF antibody titre of | |
|---|---|---|---|
| Antigen | Immune serum | Immune serum | Normal mouse/ rat serum |
| FBJ 14/3 | Mouse anti-FBJ 11/4 | 16 | <4* |
| FBJ 14/3 | Mouse anti-MSV-H | <4* | <4* |
| CH 3/9 | Mouse anti-MSV-H | 16 | <4* |
| CH 3/9 | Mouse anti-FBJ 11/4 | <4* | <4* |
| CH 2/5 | Mouse anti-MSV-H | 16 | <4* |
| CBA spleen | Mouse anti-FBJ 11/4 | <4* | <4* |
| CBA spleen | Mouse anti-MSV-H | 0 | <4* |
| CBA muscle | Mouse anti-FBJ 11/4 | 0 | 0 |
| CBA muscle | Mouse anti-MSV-H | 0 | 0 |
| FBJ 11/6 | Rat anti-GLV | 16 | 0 |
| FBJ 13/4 | Rat anti-GLV | 32 | 0 |
| CH 2/5 | Rat anti-GLV | 32 | 0 |
| CH 3/9 | Rat anti-GLV | 16 | 0 |

CH, sarcomata induced by MSV-H in CBA mice.
* <4 denotes trace of complement consumption in first well.

*Presence of complement fixing antigen in FBJ sarcoma cells reactive with naturally occurring murine antibody with Gross (G) specificity*

A naturally occurring antibody in the serum of aged ex-breeding female C57B1 mice was also demonstrated by complement fixation tests. This antibody was found to react with antigens prepared from normal AKR spleen, GLV-induced rat lymphoma (ASL–4) and FBJ sarcomata (FBJ 11/6, 13/4 and 14/3) with reciprocal titres in the range 16–32 and were invariably reactive to one dilution

TABLE VII.—*Reactivity of Naturally Occurring CF Antibody in the Serum of Aged Ex-breeding C57Bl/6 Females with Antigens of Murine Lymphoid Tissues and MSV-induced Tumours*

| Source of | | Reciprocal CF antibody titre of | |
|---|---|---|---|
| Antigen | Immune serum | Immune serum | Normal mouse serum |
| AKR-spleen | Mouse anti-FBJ 7/6 | 8 | 0 |
| AKR-spleen | Aged C57Bl mouse antiserum | 32 | 0 |
| AKR-spleen | Young C57Bl mouse serum | 4 | 0 |
| ASL-4 primary | Mouse anti-FBJ 11/4 | 8 | 0 |
| ASL-4 primary | Aged C57Bl mouse antiserum | 16 | 0 |
| FBJ 11/6 | Aged C57Bl mouse antiserum | 16 | 0 |
| FBJ 13/4 | Aged C57Bl mouse antiserum | 16 | 0 |
| FBJ 14/3 | Aged C57Bl mouse antiserum | 32 | <4* |
| CH 2/5 | Aged C57Bl mouse antiserum | 4 | <4* |
| CH 3/9 | Aged C57Bl mouse antiserum | 4 | <4* |
| CBA spleen | Aged C57Bl mouse antiserum | 4 | <4* |
| CBA muscle | Aged C57Bl mouse antiserum | <4* | 0 |

CH = sarcomata induced by MSV-H in CBA mice.
* <4 denotes trace of complement consumption in first well.

greater than hyperimmune FBJ antisera (Table VII). By contrast, the antibody failed to fix complement in the presence of similar preparations from MSV-H induced sarcomata or normal CBA spleen and muscle, reciprocal titres falling in the range 0–4. Furthermore, sera from virgin C57Bl mice less than 10 months old were unreactive in this test system. This antibody thus displayed preferential reactivity with Gross-positive tissues, as distinct from FMR virus infected tissues and demonstrated common antigens on AKR lymphoid and FBJ sarcomatous tissues.

### DISCUSSION

Murine C-type RNA tumour viruses specify a complex of virion and cellular antigens. These have been the subject of intensive study, particularly in relation to the antigens of Gross (G) or " wild " type murine leukaemia virus (MLV). Classification of various specificities has been made possible by the production of anti-sera in heterologous and homologous hosts and the use of naturally occurring antibodies in certain strains (*e.g.* C57Bl) highly resistant to MLV oncogenesis (Aoki *et al.*, 1972). To examine the relationship of the FBJ viruses to Gross or " wild " type leukaemias and to another MSV isolate, MSV-H, we have used rat and mouse antisera with CF activity to antigens specified by viruses of the MLV–MSV complex.

An antigen common to murine C-type RNA tumour viruses has been demonstrated by various serological techniques, including immunodiffusion (Geering, Old and Boyse, 1966) complement fixation (Hartley *et al.*, 1965) and immunofluorescence (Lejneva and Abelev, 1970) and has been shown to be an internal component of the virus particle (Schafer *et al.*, 1969). For the detection of this antigen, termed the species or group specific (gs) antigen (Gilden and Oroszlan, 1971), sera were raised in heterologous hosts bearing neoplasms induced by the murine agents or by immunization with disrupted virus particles. The former method evokes a complex humoral response involving antibodies to several antigenic specificities including gs and virion envelope antigens (VEA) (Herberman, 1972) as well as cell surface components, such as Gross cell surface antigens (GCSA), and type specific non-virion antigens determined by the viral genome on the surface of Gross virus infected cells (Stockert, Old and Boyse, 1971).

In this study, all tumours induced by MSV-FBJ were positive in the CF test using sera raised in our own laboratory from AS rats bearing transplants of GLV induced lymphomata and with reference AKR rat antisera obtained from the National Institutes of Health, U.S.A. Similar CF reactivity was observed against antigen prepared from cells transformed by the FMR subgroup sarcoma virus (MSV-H), from primary AKR mouse lymphomata and from the immunizing GLV-induced lymphoma. These antisera raised against Gross and AKR viruses undoubtedly contain antibodies to VEA of the G subgroup as well as other specificities since they effectively neutralize virus and react with membrane expressed VEA, as assessed by indirect IF tests (to be published). However, the spectrum of CF activity with the rat antisera indicates that the shared specificity of G and FMR neoplasms is predominantly that of the gs antigen of the murine leukaemia–sarcoma virus complex. Confirmation of this conclusion has come from immunofluorescence studies on the localization of gs antigen in acetone fixed monolayers of cells transformed *in vivo* by MSV-FBJ and MSV-H (unpublished data). No attempt was made in this study to distinguish between the various subgroup specific antigens (gs 1, 3).

Immunization of homologous hosts with MLV-MSV antigens evokes a humoral response to a different though related antigenic spectrum. Since mice are completely tolerant to the gs antigen, antibodies to the internal component of the virus are not produced. Antibodies may, however, be produced to VEA and GCSA as well as to alloantigenic specificities. In this study, the evocation of alloantibodies was precluded by the use of strictly syngeneic recipients for immunization against FBJ transformed cells. Sera raised by this protocol permitted distinction between virus induced neoplasms expressing antigens of different type specificities.

All FBJ tumours were positive in the CF test with sera from mice which had received irradiated isografts of FBJ sarcomata. CF antigen was also detected in extracts of the spleens of mice bearing primary FBJ induced sarcomata. CF antibody titres were lowest, and occasionally indetectable, in mice bearing tumour transplants, which is suggestive of the formation of antigen–antibody complexes. Whether the antibodies detected with the homologous sera are to VEA or GCSA, or both antigens, was not resolved in this study. Sera from FBJ tumour bearers are virus neutralizing (Kelloff *et al.*, 1969) which is indicative of anti-VEA antibodies. The coexistence of antibodies with specificity for GCSA cannot, however, be excluded. Studies undertaken in parallel by membrane immunofluorescence have shown that the antibodies evoked are essentially to membrane expressed antigens (to be published).

Consistent with the interpretation that hyperimmune FBJ sera contain anti-VEA antibodies (and probably anti-GCSA antibodies), antigens extracted from normal AKR lymphoid tissues in which Gross leukaemia virus is indigenous, were active in the CF test. Of additional interest was the expression of this CF antigen in a radiogenic myeloid leukaemia of RF mice, transmissible in cell-free extracts and therefore putatively of viral aetiology. Positive reactivity with sera exhibiting specificity for Gross virus related antigens suggests that the putative agent is also of " wild " type subgroup, although antigenic conversion of this long transplanted line is a possibility which cannot be excluded. By contrast, CF tests undertaken with extracts from a spontaneous A strain leukaemia, for which a viral aetiology has not been established (Marchant, personal communication) were negative.

Further specificity tests with antigens prepared from primary AKR lymphomata confirmed the Gross type specificity of the CF antigen reactive with hyperimmune FBJ sera. By contrast, antigenic distinction from MSV-H was established in reciprocal CF tests with antisera prepared in the respectively immunized donors and antigens from the corresponding tumour tissues. MSV-H is antigenically indistinguishable from other leukaemic and sarcomagenic agents of the FMR subgroup, as demonstrated by transplantation tests, serological techniques and *in vitro* cell mediated cytotoxicity (Chuat *et al.*, 1969). The lack of antigen cross reactivity by CF emphasizes the separate categories of the MSV isolates, MSV-FBJ and MSV-H, and the uniqueness of the former as the only known " wild " type sarcomagenic virus.

Further data on the Gross type specificity of FBJ sarcoma cells and the nature of antigens expressed by them were derived from CF tests undertaken with sera from aged exbreeding C57Bl female mice which have been shown to develop antibody of G specificity (Aoki *et al.*, 1966) but without demonstrable virus neutralizing activity. Immunoelectron microscopic studies (Aoki *et al.*, 1970) confirmed that the antigen detected by the naturally occurring G antibody was a cellular antigen and not a constituent of the virion. In the present study, reactivity of C57Bl antisera closely paralleled that of hyperimmune FBJ sera, indicating that cellular and virion specificities are simultaneously present in FBJ sarcoma cells. Moreover, the reactivity of the C57Bl antibody by CF was comparable with that detected in the same sera by membrane immunofluorescence on AKR lymphoid and FBJ sarcoma cells, suggesting that the antibodies detected by the two methods may be identical. It is possible that CF antigen represents exfoliated GCSA. Equally tenable, however, are the alternatives that the CF antibody present in C57Bl antiserum reacts with G soluble antigen (GSA) sharing a common specificity with GCSA (Aoki *et al.*, 1972), or that these antisera contain G antibodies of more than one specificity.

Whilst there are likely to be other virus associated antigens present on cells replicating FBJ viruses, these data confirm the Gross-type specificity of FBJ sarcomata and indicate the presence of G type-specific cellular (GCSA) and virion antigens (VEA) in cells transformed by this agent.

We thank Dr N. W. Nisbet, Director of Research, for his support, and Miss Heulwen Jones for secretarial assistance.

This study was supported by grants from the Medical Research Council and the Cancer Research Campaign.

### REFERENCES

AOKI, T., BOYSE, E. A. & OLD, L. J. (1966) Occurrence of Natural Antibody to the G (Gross) Leukemia Antigen in Mice. *Cancer Res.*, **26**, 1415.

AOKI, T., BOYSE, E. A., OLD, L. J., DE HARVEN, E., HAMMERLING, U. & WOOD, H. A. (1970) G (Gross) and H-2 Cell-surface Antigens: Location on Gross Leukemia Cells by Electron Microscopy with Visually Labelled Antibody. *Proc. natn. Acad. Sci. U.S.A.*, **65**, 569.

AOKI, T., HERBERMAN, R. B., JOHNSON, P. A., LIU, M. & STURM, M. M. (1972) Wild-type Gross Leukemia Virus: Classification of Soluble Antigens (GSA). *J. Virol.*, **10**, 1208.

CHUAT, J. C., BERMAN, L., GUNVÉN, P. & KLEIN, E. (1969) Studies on Murine Sarcoma Virus: Antigenic Characterisation of Murine Sarcoma Virus Induced Tumour Cells. *Int. J. Cancer*, **4**, 465.

FINKEL, M. P., BISKIS, B. O. & JINKINS, P. B. (1966) Virus Induction of Osteosarcoma in Mice. *Science, N.Y.*, **151**, 698.

GEERING, G., OLD, L. J. & BOYSE, E. A. (1966) Antigens of Leukemias Induced by Naturally Occurring Murine Leukemia Virus: their Relation to the Antigens of Gross Virus and Other Murine Leukemia Viruses. *J. exp. Med.*, **124**, 753.

GILDEN, R. V. & OROSZLAN, S. (1971) Structural and Immunologic Relationships among Mammalian C-type Viruses. *J. Am. vet. med. Ass.*, **158**, 1099.

HARTLEY, J. W., ROWE, W. P., CAPPS, W. I. & HUEBNER, R. J. (1965) Complement-fixation and Tissue Culture Assays for Mouse Leukemia Viruses. *Proc. natn. Acad. Sci. U.S.A.*, **53**, 931.

HARVEY, J. J. (1964) An Unidentified Virus which Causes the Rapid Production of Tumours in Mice. *Nature, Lond.*, **204**, 1104.

HARVEY, J. J. & EAST, J. (1971) The Murine Sarcoma Virus (MSV). *Int. Rev. exp. Path.*, **10**, 265.

HERBERMAN, R. B. (1972) Serological Analysis of Cell Surface Antigens of Tumors Induced by Murine Leukemia virus. *J. natn. Cancer Inst.*, **48**, 265.

JENKINS, V. K. & UPTON, A. C. (1963) Cell-free Transmission of Radiogenic Myeloid Leukemia in the Mouse. *Cancer Res.*, **23**, 1748.

JONES, D. B. & MOORE, M. (1973) Tumour-associated Transplantation Antigens of Neoplasms Induced by a Naturally Occurring Murine Sarcoma Virus (FBJ-MSV). *Br. J. Cancer*, **27**, 415.

KELLOFF, G. J., LANE, W. T., TURNER, H. C. & HUEBNER, R. J. (1969) *In vivo* Studies of the FBJ Murine Osteosarcoma Virus. *Nature, Lond.*, **223**, 1379.

LEJNEVA, O. M. & ABELEV, G. I. (1970) Immunofluorescent Study of the Group-specific Antigen of Murine Leukaemia Viruses. *Int. J. Cancer*, **6**, 153.

LEVY, J. A., HARTLEY, J. W., ROWE, W. P. & HUEBNER, R. J. (1973) Studies of FBJ Osteosarcoma Virus in Tissue Culture. I. Biologic Characteristics of the " C " Type Viruses. *J. natn. Cancer Inst.*, **51**, 525.

MOORE, M. & WILLIAMS, D. E. (1972) Studies on the Antigenicity of Radiation-induced Murine Osteosarcomata. *Br. J. Cancer*, **26**, 90.

PRICE, C. H. G., MOORE, M. & JONES, D. B. (1972) FBJ Virus-induced Tumours in Mice. A Histopathological Study of FBJ Virus Tumours and their Relevance to Murine and Human Osteosarcoma Arising in Bone. *Br. J. Cancer*, **26**, 15.

RHIM, J. S., HUEBNER, R. J., LANE, W. T., TURNER, H. C. & RABSTEIN, L. (1969) Neoplastic Transformation and Derivation of a Focus-forming Sarcoma Virus in Cultures of Rat Embryo Cells Infected with a Murine Osteosarcoma (FBJ) Virus. *Proc. Soc. exp. Biol. Med.*, **132**, 1091.

SCHAFER, W., ANDERER, F. A., BAUER, H. & PISTER, L. (1969) Studies on Mouse Leukemia Viruses. I. Isolation and Characterisation of a Group-specific Antigen. *Virology*, **38**, 387.

SEVER, J. L. (1962) Application of a Microtechnique to Viral Serological Investigations. *J. Immun.*, **88**, 320.

STOCKERT, E. A., OLD, L. J. & BOYSE, E. A. (1971) The $G_{IX}$ System. A Cell Surface Allo-antigen Associated with Murine Leukemia Virus; Implications regarding Chromosomal Integration of the Viral Genome. *J. exp. Med.*, **133**, 1334.

YUMOTO, T., POEL, W. E., KODAMA, T. & DMOCHOWSKI, L. (1970) Studies on the FBJ Virus-induced Bone Tumors in Mice. *Tex. Rep. Biol. Med.*, **28**, 145.